# Estrogen and inflammation modulate estrogen receptor alpha expression in specific tissues of the temporomandibular joint

**DOI:** 10.1186/1477-7827-7-155

**Published:** 2009-12-31

**Authors:** Jyoti Puri, Bob Hutchins, Larry L Bellinger, Phillip R Kramer

**Affiliations:** 1 Department of Biomedical Sciences, Texas A&M Health Science Center, Baylor College of Dentistry, Dallas, Texas, USA

## Abstract

**Background:**

Estrogen is known to play role in temporomandibular joint (TMJ) disorders and estrogen effects can be mediated by estrogen receptor (ER) alpha present in the TMJ. Cells expressing the estrogen receptor ERalpha are present in the temporomandibular joint (TMJ) but changes in expression due to estrogen and inflammation have not been characterized. In this study, ERalpha protein content and the number of cells expressing ERalpha was measured in 17 beta-estradiol-treated rats after inflammation was induced in the TMJ.

**Methods:**

Sixteen ovariectomized female rats were divided into two groups such that one group received 17 beta estradiol (E2) and the other was given vehicle (VEH). Groups were then subdivided further, one received injections of saline and the other received Complete Freund's adjuvant (CFA) within the superior joint space of the TMJ. Thus the four groups include no E2/saline, E2/saline, no E2/CFA and E2/CFA. After treatment, the rats were sacrificed, and the TMJ anterior, disc, retrodiscal and synovial tissues were analyzed by western blot and immunocytochemistry. Positive stained cells were counted using a Nikon epifluorescent microscope.

**Results:**

The western blot showed that ERalpha protein significantly decreased with inflammation. The number of ERalpha-positive cells in the TMJ was not affected by inflammation or 17 beta-estradiol with exception of the retrodiscal tissue. In the retrodiscal tissue 17 beta-estradiol significantly decreased the number of ERalpha-positive cells but only in a non-inflamed joint.

**Conclusions:**

In conclusion, inflammation and 17 beta-estradiol can modulate ERalpha expression in the TMJ but the effects are tissue specific.

## Background

Epidemiological studies have shown that during their reproductive years, women experience a higher frequency of temporomandibular joint disorders than males with a ratio of = 1.5- 2 [1]. It has been postulated that estrogen could be the primary molecule leading to the higher frequency of TMJ disorders in women. Endogenous estrogen affects the remodeling processes within the TMJ [2,3] possibly by changing the extracellular matrix in the joint [4] or by changing bone volume [2]. Such changes can result in internal derangement of the TMJ. Estrogen modulation of matrix metalloproteinase (MMP) expression has also been observed in the cartilage of the TMJ [5,6]; high levels of MMP have been reported to play a role in TMJ osteoarthritis [7,8]. Moreover, the level of estrogen in the synovial fluid has been correlated to the severity of arthritis in postmenopausal women [9], and in animal models estrogen has been shown to modulate inflammation in the TMJ [10,11].

17 β-estradiol is the most potent and dominant form of estrogen in the human female, having biological effects through binding the estrogen receptor alpha (ERα) and beta (ERβ) [12]. ERα and ERβ acts via binding to the DNA [13] or by having biological effects through interacting with various kinases, such as mitogen-activated protein kinase, phosphatidylinositol-3 kinase or tyrosine kinase [12]. Also both receptors can interact with membrane-associated molecules such as ion channels and G protein-coupled receptors to alter the physiology of the cell [12].

The wide distribution of the estrogen receptors ERα and ERβ in the thymus [14,15], bone marrow [16] and spleen [17] suggests that it plays a modulatory role in the immune system, such as modulating differentiation, activation, proliferation and antibody production from lymphoid cells [18]. Immune responses are important in joint disorders and an alteration in ERα and ERβ expression would have an impact on how estrogen affects the immune response in the TMJ.

In particular, ERα was found in the articular cartilage and subchondral bone of TMJ which implies that estrogen directly acted on the cells in the TMJ tissue to alter the gene expression and cellular physiology [19]. In contrast to the ERα expression in the rat, ERα nuclear staining was not detected in the bone, cartilaginous or connective tissues of the human or baboon TMJ, but it was present in the immune cells such as macrophage and lymphocytes [20,21].

In addition ER knockout studies have shown a major role for ERα in immune modulation [22,23]. Because ERα is present in the immune cells in the human TMJ [20,21], a likely target of 17 β-estradiol in the human TMJ is ERα-positive immune cells. Furthermore ER-α gene polymorphisms is associated with a predisposition to TMJ disorders [24]. Such data from both animal and clinical studies support a significant role of ERα in the increased incidence of TMJ inflammation [24,25].

In an intact rat 17 β-estradiol and progesterone concentrations change during the estrus cycle [26]. Multiple fluctuating hormones could complicate our understanding of how 17 β-estradiol modulates estrogen receptor expression. Hormone administration in overiectomized animals allows us to study the effect of 17 β-estradiol, avoiding fluctuating levels. Thus, our study was focused on the effect of a single, physiological concentration of 17 β-estradiol. This concentration of 17 β-estradiol was equal to that observed during the estrus cycle [26].

The level of receptor will determine the cellular sensitivity to 17 β-estradiol, because this hormone acts primarily through the estrogen receptor(s) [27]. This paper particularly focuses on ERα expression in response to17β-estradiol and inflammation. The aim of the present study is to first quantify the tissue content of ERα in the female rat TMJ, and second, to determine whether changes in the ERα expression in the TMJ result from alterations in inflammation or the concentration of 17β-estradiol.

## Methods

### Animals and tissue preparation

The Baylor College of Dentistry Institutional Animal Care and Use Committee approved the experimental protocol. Female Sprague Dawley rats (~230 gm) were ovariectomized by Harlan Industries, Houston TX. Upon arrival, the rats were housed individually and kept on a 14:10 h light/dark cycle with lights on at 0600 h. All the rats were anesthetized with ketamine (45 mg/kg) and rompun (4.8 mg/kg). Using sterile technique, the rats were implanted with primed, 14-day Alzet mini-osmotic pumps dispensing vehicle, polyethylene glycol (no estrogen replacement control group) or 750 ng/day of 17β-estradiol benzoate. These animals were then subdivided further; one group received bilateral injections of 0.9% saline (30 μl) and the other group received an injection of 30 μg (30 μl) of complete Freund's adjuvant (CFA) within the superior joint space of the TMJ just above the disc. The animals were removed from their cages and sacrificed with an overdose of CO_2 _after 48 h. The animals were decapitated; blood collected and the heads were placed in plastic bags and submerged in ice water. For western analysis, the TMJ anterior, disc, retrodiscal and synovium were dissected from one side by performing a superficial, horizontal skin incision parallel and just inferior to the zygomatic arch. The masseter and temporalis muscles were cut away from the arch and the ramus of the mandible so that the arch could be removed with ronjeurs. The neck of the condyle was grasped with hemostats and the condylar neck fractured to allow removal of any remaining musculature and access to the anterior, disc, retrodiscal and synovial tissues. Dissected TMJ tissue was excised from the posterior neck of the condyle, and all anterior tissue, articular disc, retrodiscal tissue and synovium were removed, placed in liquid nitrogen and stored at -80°C. For histological analysis, a 0.5 cm cube of TMJ tissue centered on the condyle was removed, en bloc, from the other side and placed in 4% paraformaldehyde at 4°C overnight. The tissue was then decalcified in 10% EDTA for 2 weeks, embedded in paraffin wax and 8 μm sections were cut and mounted on Superfrost glass slides (Fisher Scientific, Pittsburgh, PA) for immunohistochemical or hematoxylin and eosin staining.

### Quantitation of hormone levels

17 β-estradiol was quantitated in the blood plasma by first, vigorously mixing 0.5 ml of plasma with 5 ml of ether. Second, the solution was frozen in an ethanol, dry ice bath and the ether phase decanted. The ether phase contains the lipid soluble hormones. The ether was evaporated and the remaining residue suspended in RIA buffer supplied by the manufacturer. The concentration of 17 β-estradiol was quantitated following the manufactures directions (ICN Biomedicals, Costa Mesa, CA).

### Western blot analysis

The previously isolated, frozen TMJ anterior, disc, retrodiscal and synovial tissues were homogenized in ice-cold protein-extraction buffer (75 mM potassium acetate pH 7.4, 300 mM NaCl, 10 mM EDTA, .25% Triton X-100, protease inhibitors), centrifuged at 7500 g for 15 min at 4°C, and the resulting supernatant was collected and stored at -20°C. Each sample was tested for protein content by standard BCA assay (Thermo Fisher Scientific, Rockford, IL); 10 μg of total protein was loaded onto a SDS PAGE, separated by electrophoresis, transferred to a membrane, and the membrane was blocked with 1.5% v/v BSA in TBS-T (50 mM Tris-HCl pH 7.5, 150 mM NaCl, 0.05% Tween 20) for 2 h at room temperature. After blocking, the ERα mouse monoclonal antibody (Thermo Fisher Scientific) was added at a dilution of 1:1000 overnight at 4°C. The membrane was then incubated for 2 h with a goat anti-mouse secondary antibody conjugated to horseradish peroxidase. The products were detected by means of an enhanced chemiluminescence light detection kit (Bio-Rad, Hercules, CA). After detection of the ERα band the blot was stripped according to the manufactures directions with Erase-It (Thermo Fischer Scientific, Rockford, IL) and re-probed with a β-actin antibody (Cell Signaling, Boston, MA) at a dilution of 1:000 followed by addition of a goat anti-rabbit secondary, detection and development of the film. The BioMax films (Kodak, Rochester, New York) were developed, digitally scanned, and the integrated optical density of the resulting bands was collected and analyzed using Scion Image (Scion Corporation, Frederick, MD).

### Immunocytochemistry

The paraffin wax was removed from the tissue sections, and then the sections were processed for staining with hematoxylin and eosin or for immunohistochemistry. Light microscope images were captured by Zeiss Axioplan. Immunohistochemistry was completed by first quenching endogenous peroxidase with 0.3% H_2_O_2 _in PBS for 30 min, blocking in 10% normal goat serum and PBS for 1 h and then incubating the slide with two primary antibodies; mouse anti-ERα antibody diluted to 1:250 (Thermo Fisher Scientific) and goat polyclonal CD14 (Santa Cruz Biotechnology) diluted to 1:100 overnight at 4°C. The sections were subsequently treated with two fluorescent conjugated secondary antibodies (goat anti-mouse Alexa 488 (Invitrogen, Carlsbad, CA) at 1:2000 dilution and rabbit anti-goat Alexa 633 in PBS at 1:500 (Molecular Probes) for an hour at room temperature. The sections were rinsed and then cover-slipped with fluoromount-G medium (Southern Biotech, Birmingham, AL) containing DAPI counterstain. The total (DAPI positive) and ERα positive cells (Alexa 488 positive) were counted on every 10^th ^section to obtain a representative sample through the entire TMJ region [28] using a Nikon epifluorescent microscope. The images were also analyzed with a Leica TCS-SP2 confocal microscope and arranged with Adobe Photoshop.

### Data and statistical analysis

The data were analyzed using two-way ANOVA using the factors of hormone and inflammation. Data that showed a significant group effect were further analyzed using a Bonferroni post-hoc test. P-values of < 0.05 were considered to be statistically significant. The results were expressed as the mean and standard error of the mean (SEM).

## Results

**Figure 1:**
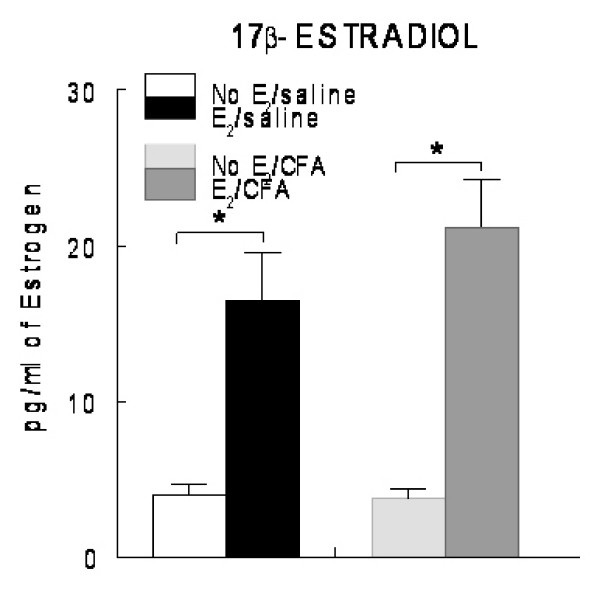
**Concentration of 17 β-estradiol in the plasma of ovariectomized female rats**. Ovariectomized (OVX) rats were implanted with Alzet pumps dispensing 750 ng/day of E_2 _or the same volume of the vehicle polyethylene glycol (no E_2_). OVX rats infused with no E_2 _or E_2 _were given a 30 μl intra-articular temporomandibular joint (TMJ) injection of saline or 30 μg complete Freund's adjuvant (CFA) seven days after pump implantation. Treatment groups consisted of OVX rats having their TMJ injected with saline (No E_2_/saline); OVX rats supplemented with E_2 _that had saline injected into their TMJ (E_2_/saline); OVX rats injected with CFA (No E_2_/CFA) and OVX rats supplemented with E_2 _that had CFA injected into the TMJ (E_2_/CFA). 48 h following TMJ injection the animals were sacrificed and the concentration of E_2 _was measured in the plasma by radio-immunoassay. The histogram shows the mean ± SEM concentration of 17 β-estradiol per ml of plasma from the pooled values of four animals per treatment group, * *p *< 0.05.

In the rat, Alzet pumps released 750 ng/day of 17 β-estradiol benzoate. This amount produced a plasma 17 β-estradiol concentration of approximately 20 pg/ml (Fig. 1), a concentration that was significantly higher than ovariectomized rats receiving vehicle and was similar to the amount of plasma 17 β-estradiol reported for rats during the estrus cycle [26].

**Figure 2:**
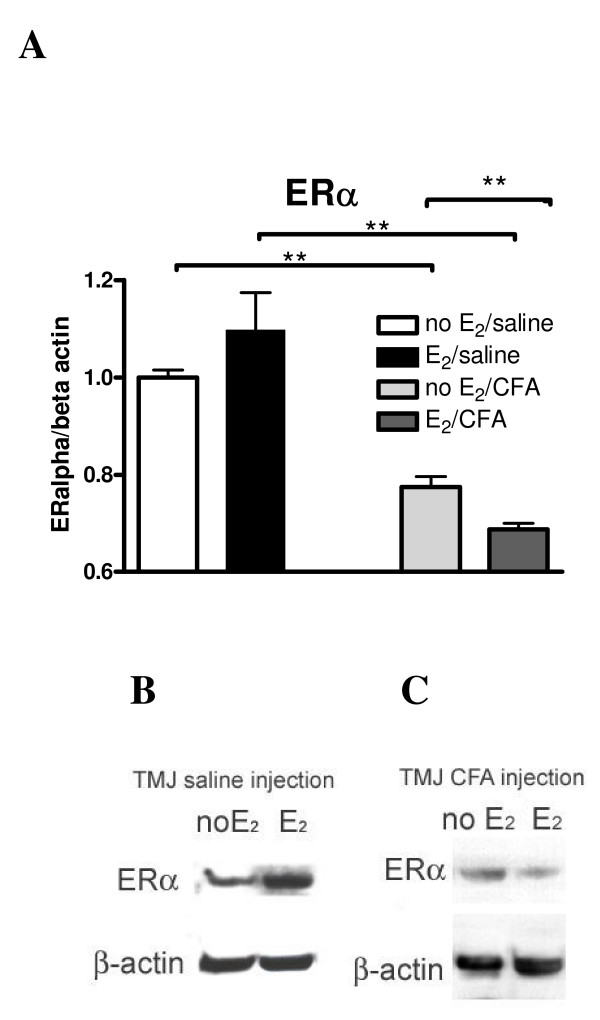
**Western blot of protein isolated from the temporomandibular joint (TMJ) of ovariectomized female rats treated with 17 β-estradiol**. Ovariectomized (OVX) rats were given a physiological dose of (E_2_) or vehicle (No E_2_) using osmotic pumps and after 7 days of estrogen treatment the TMJ was injected with 30 μl of saline or 30 μg CFA. Protein from the TMJ anterior, disc, retrodiscal and synovial tissues was isolated, 10 μg of protein was loaded in each lane of the gel and the western blot was probed with anti-ERα and then anti-β-actin. (A) Optical densities of the ERα specific band (65 kDa) were determined from the western blot and the optical density of the ERα specific band was normalized to the β-actin band. The normalized mean ± SEM optical density values are shown in the histogram. Each lane in panels B and C is a representative sample from one animal in each treatment group. ** = p < 0.01.

After performing a western blot on the samples from the TMJ no significant change in ERα expression was found due to administration of 17 β-estradiol in either the saline (Fig 2A and 2B) or CFA (Fig. 2A and 2C) injected group. The amount of ERα protein significantly decreased after injection of CFA into both vehicle or 17 β-estradiol treated rats (Fig. 2A).

**Figure 3:**
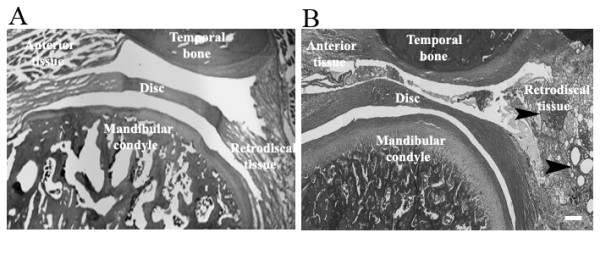
**Histology of sagittal section of a female rat temporomandibular joint**. (A) Representative image of a vehicle treated OVX rat 48 h after injecting 30 μl of saline. (B) Representative image of a vehicle-treated ovariectomized (OVX) rat 48 h after injecting 30 μl of complete Freund's adjuvant in the upper joint space of the temporomandibular (TMJ). The TMJ was removed en bloc 48 h after injection, paraffin-embedded, sectioned at 8 μm and stained with hematoxylin and eosin. Sight of inflamed tissue is indicated by black arrowheads. Size bar = 500 μm

**Figure 4:**
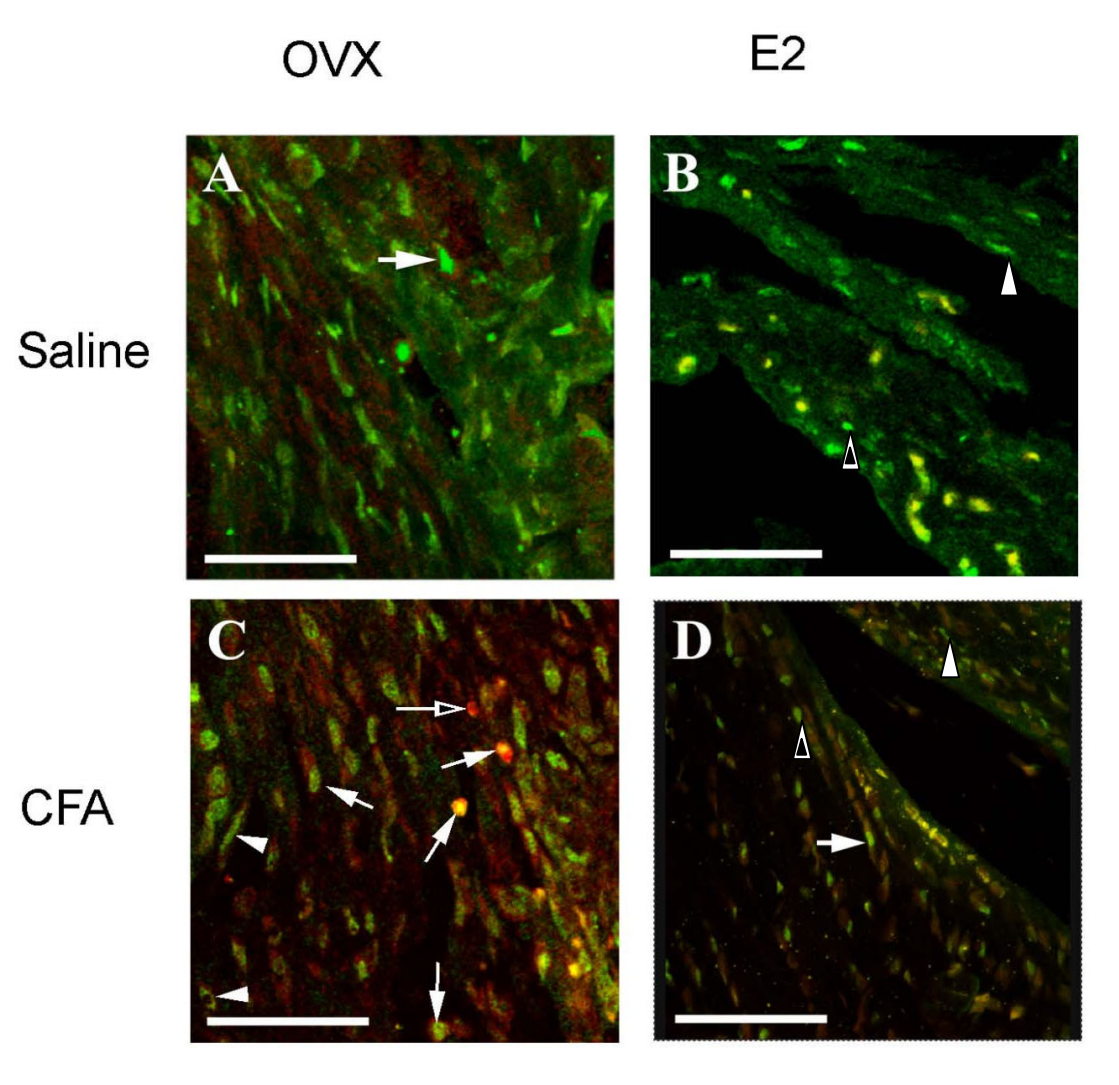
**Immunofluorescent staining of ERα and CD14 observed in the retrodiscal tissue of the TMJ in all four treatment groups**. A) No E_2_/saline, B) No E_2_/CFA, C) E_2_/saline, D) E_2_/CFA. The sections were immunohistochemically stained with mouse anti-ERα and goat polyclonal anti- CD14 primary antibodies followed by anti-mouse Alexa 488 conjugated (green) and anti-goat Alexa 633 conjugated (red) secondary respectively. ERα nuclear with CD14 cytoplasmic staining (arrows), ERα cytoplasmic staining (arrowhead), ERα nuclear staining (open arrowhead) and CD14 staining (open arrow) were observed. Size bar for A, B, D = 47.5 μm.; C = 50 μm.

Swelling was observed in the retrodiscal tissue of ovariectomized rats injected with CFA (Fig. 3B). This was in contrast to the retrodiscal tissue of saline injected rats that had no swelling (Fig. 3A). Similar levels of swelling were observed in ovariectomized rats injected with vehicle or 17 β-estradiol that were then injected with CFA (data not shown). Nuclear and cytoplasmic ERα immunostaining was observed in the highly vascularized retrodiscal tissue (Fig. 4) and in the synovial lining cells. Double stained ERα and CD14 positive cells were found in the retrodiscal area (Fig. 4) and synovium of all four groups. These double labelled cells were observed more in CFA injected group as compared to saline group. When the primary antibody was not added (negative control), no signal was observed (data not shown).

**Figure 5:**
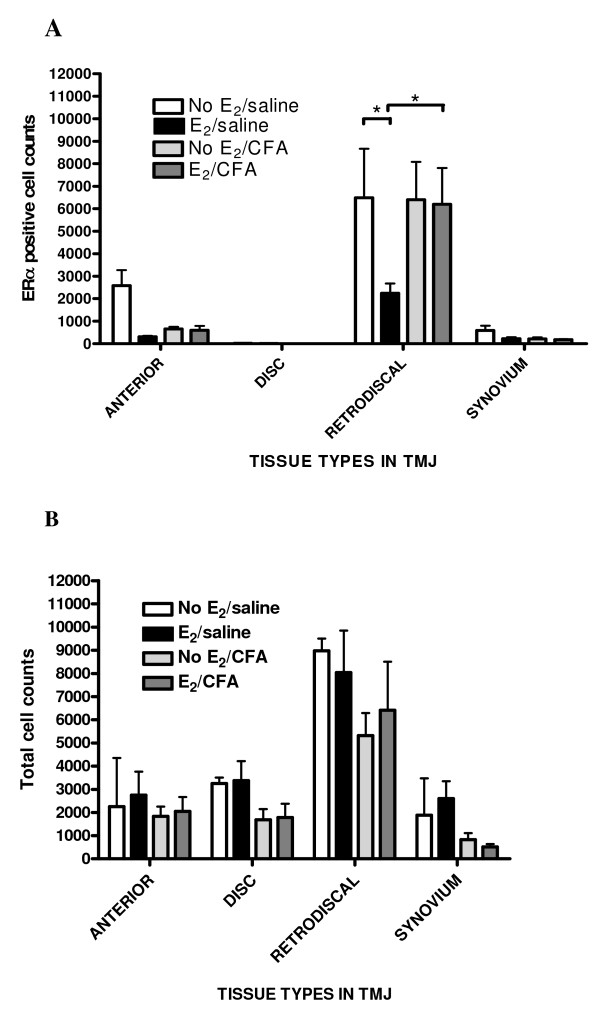
**Cell counts within the TMJ**. Quantitation of (A) ERα-containing cells and (B) the total number of cells within the temporomandibular joint (TMJ). Rats were given a constant infusion of vehicle (No E_2_) or 17 β-estradiol (E_2_) using osmotic pumps for one week, and then the TMJ was injected with saline or 30 μg of CFA to produce four treatment groups: No E_2_/saline, No E_2_/CFA, E_2_/saline, E_2_/CFA. The TMJ was removed en bloc 48 h after TMJ injection, paraffin-embedded, sectioned at 8 μm and immunostained for ERα (Alexa 488 positive) and counterstained with DAPI. The histogram shows the number of ERα-positive stained cells in each of the four tissues (i.e., anterior, disc, retrodiscal and synovial) for each treatment group. Counts included cells with cytoplasmic and nuclear ERα staining. Following staining, the number of DAPI-positive cells in each tissue region was counted for each treatment group. Bars on the histogram were generated from the cell counts of four different animals per treatment group whose values were pooled to give a mean ± SEM. * = p < 0.05.

ERα-positive cells were counted in the anterior, disc, retrodiscal and synovial tissue, but few ERα-positive cells were observed in the disc (Fig. 5A). Of the total cells observed, the counts showed that 84% of the ERα-positive stained cells were in the retrodiscal tissue, 13% of the cells were in the anterior tissue and 3% in the synovium. 17 β-estradiol (E_2_) treatment significantly decreased the number of cells expressing ERα in the retrodiscal tissue of rats with a non-inflamed TMJ (No E_2_/saline vs. E_2_/saline; Fig. 5A). The number of cells expressing ERα in the inflamed retrodiscal tissue of the TMJ was unchanged after giving the rats 17 β-estradiol (No E_2_/CFA vs. E_2_/CFA). In the retrodiscal tissue, CFA significantly increased the number of ERα positive cells in those rats receiving 17 β-estradiol (E_2_/saline vs. E_2_/CFA) but not in rats given vehicle (No E_2_/saline vs. no E_2_/CFA; Fig. 5A). (Though there was a trend showing a higher number of cells in the No E_2 _saline group, the number of cells expressing ERα did not significantly change in the anterior, disc or synovium of the TMJ as a result of giving 17 β-estradiol or CFA (Fig. 5A). Also, the total number of cells in these tissues did not change following 17 β-estradiol or CFA treatment (Fig. 5B).

## Discussion

In these studies we administered 17 β-estradiol to ovariectomized rats such that the plasma concentration of hormone was physiological and similar to the concentration observed during the rats estrus cycle [26]. In ovariectomized rats, treated with and without 17β-estradiol, injection of CFA into the TMJ induced swelling of the retrodiscal tissue. Induction of inflammation decreased the expression of ERα in the TMJ of rats injected with and without 17β-estradiol. 17 β-estradiol reduced the number of ERα positive cells in the retrodiscal tissue but only in animals that did not have an inflammation, suggesting first that 17 β-estradiol acts in a tissue specific manner in the TMJ region and second that the inflammation can alter the effect of estradiol.

Knowing that on western blots estrogen receptor levels decreased in the TMJ region after injection of CFA, the question arose as to whether this decrease was due to a reduction in the number of cells expressing the receptor or to a decrease in the number of receptors per cell. For this reason we counted the number of cells expressing ERα. In the anterior and synovial tissue the number of ERα positive cells in the No E_2_/saline group trended (showed a trend to be) higher than in the No E_2_/CFA group, suggesting the CFA induced a decrease in ERα levels, consistent with the western results. Animals receiving 17 β-estradiol had a decreased amount of ERα on a western blot after CFA injection, but the number of ERα positive cells increased in the anterior and retrodiscal tissue. Whereas in the synovium the number of ERα positive cells did display a downward trend after CFA injection. Double stained ERα and CD14 positive immune cells observed in the retrodiscal area and synovium of all four groups suggest the presence of ERα positive immune cells consistent with the ERα-positive immune cells found in humans [20]. Another study also shows that the synovium is highly populated with monocytes and macrophages [29]. Macrophage-like and fibroblast-like synoviocytes stain for both ERα and ERβ [30-32]. Although few in number, the ERα positive cells in the synovium might have much higher amounts of ERα per cell than the retrodiscal tissue. In the event that a majority of the ERα protein from the TMJ was derived from the synovium we would see a decrease in ERα after CFA injection, consistent with our western blot results. Our results showing that different tissues had a different response to 17 β-estradiol was consistent with studies done on rat uterus, demonstrating the upregulation of ERα in the stromal cells and its concurrent downregulation in the epithelia of the uterus in response to estradiol [33]. Expression of the immune complex protein C3 was shown to be upregulated in the uterine epithelial cells in response to estradiol whereas expression in the liver was unaffected by the same concentration of estrogen [34]. In joint tissue 17 β-estradiol alters proteoglycan metabolism differently depending on location [35].

In the retrodiscal tissue the number of ERα positive cells increased or remained unchanged after CFA injection but the amount of ERα in each cell may have decreased leading to a reduction in the ERα observed on the western blot. To determine whether an increase in the expression per cell results in a higher expression of estrogen receptor, we needed to quantitate the fluorescent signal in each cell. Unfortunately, the florescent signal faded rapidly and we could not determine the intensity of staining per cell (i.e. ERα expression per cell) and thus, we could not determine what led to the decrease in estrogen receptor signal on the western blot.

The number of cells expressing ERα in the retrodiscal tissue was significantly lower in the 17 β-estradiol-treated rat. In humans, the estrogen receptors are present on peri-vascular immune cells (e.g., macrophages) in the TMJ [20]. 17 β-estradiol treatment has been shown to modulate gene expression in the macrophages [36-38]. In vivo studies have shown that macrophage proliferation increases with an increased estrogen concentration in female mice [39,40], implying that estrogen can increase ERα levels though stimulation of macrophage proliferation. Since we did not see an increase in ERα positive cell numbers, macrophage proliferation was not a likely cause for the increased ERα expression. Another possible explanation for the reduction in ERα expression may be that 17β-estradiol caused a down-regulation of gene expression [41] similar to previous studies that indicated 17β-estradiol inhibits ERα expression in joint tissue [42]. Interestingly, this previous study also showed that inflammation effectively blocked the inhibitory effect of 17β-estradiol, consistent with our results in the retrodiscal tissue [42].

Studies have also demonstrated decreased specific binding of radiolabeled 17β-estradiol with E2 treatment which resulted in a depletion of cytosolic ER [43,44]. Down-regulation of estrogen receptor was suggested to involve some transcriptional activation mechanisms such as proteasome-mediated proteolysis activity [45,46].

## Conclusions

A constant physiological concentration of 17 β-estradiol effects ERα expression in a non-inflamed and inflamed TMJ. But several questions remain such as what are the effects of 17 β-estradiol on ERβ in the TMJ tissues and what are the effects of progesterone or pregnancy levels of 17 β-estradiol. Future studies would need to focus on the amount of receptor per cell in different tissues as well as determine the effect of higher concentrations of 17 β-estradiol such as that occuring during pregnancy (120 pg/ml). These studies might help develop estrogenic medications for TMJ pain. Moreover, progesterone could impact estrogen receptor expression and interact with 17 β-estradiol to alter its effects.

## Competing interests

The authors declare that they have no competing interests.

## Authors' contributions

JP, BH and PRK participated in the design of the study, data collection, statistical analysis, and manuscript drafting. All authors read and approved the final manuscript.
